# Empirically supported psychodynamic psychotherapy for common mental disorders–An update applying revised criteria: Systematic review protocol

**DOI:** 10.3389/fpsyt.2022.976885

**Published:** 2022-09-15

**Authors:** Falk Leichsenring, Allan Abbass, Nikolas Heim, John R. Keefe, Patrick Luyten, Sven Rabung, Christiane Steinert

**Affiliations:** ^1^Department of Psychosomatics and Psychotherapy, University of Giessen, Giessen, Germany; ^2^Department of Psychosomatics and Psychotherapy, University of Rostock, Rostock, Germany; ^3^Department of Psychiatry and The Centre for Emotions and Health, Dalhousie University, Halifax, NS, Canada; ^4^International Psychoanalytic University (IPU), Berlin, Germany; ^5^Department of Psychiatry, Weill Medical College of Cornell University, New York, NY, United States; ^6^Faculty of Psychology and Educational Sciences, University of Leuven, Leuven, Belgium; ^7^Research Department of Clinical, Educational and Health Psychology, University College London, London, United Kingdom; ^8^Department of Psychology, University of Klagenfurt, Klagenfurt, Austria

**Keywords:** empirically supported psychodynamic psychotherapy, empirically supported psychotherapy, efficacy, effectiveness, common mental disorders

## Abstract

The approach of evidence-based medicine has been extended to psychotherapy. More than 20 years ago, criteria for empirically supported psychotherapeutic treatments (ESTs) were defined. Meanwhile a new model for empirically supported psychotherapeutic treatments has been proposed. While the empirical status of psychodynamic therapy (PDT) was assessed in several reviews using the previous criteria, the proposed new model has not yet been applied to PDT. For this reason, we will carry out a systematic review on studies of PDT in common mental disorders applying the revised criteria of ESTs. As suggested by the new model we will focus on recent systematic quantitative reviews. A systematic search for meta-analyses on the efficacy of PDT in common mental disorders will be carried out. Meta-analyses will be selected and evaluated by at least two raters along the criteria of the new proposed model. In addition, systematic reviews and individual studies addressing mechanisms of change in PDT, effectiveness under real-world conditions, cost-effectiveness and adverse events will be systematically searched for and evaluated. Finally, quality of evidence, the extent to which benefits exceed harms and strength of recommendations will be assessed per disorder using GRADE.

## Review questions

More than 20 years ago, criteria for empirically supported psychotherapeutic treatments were proposed (1, 2). For a designation as efficacious in a specific mental disorder, at least two randomized controlled trials from independent research groups were required which showed that a manual-guided treatment was superior to controls or as efficacious as an already established treatment (1). Meanwhile concerns have been raised about the applied definition of empirically supported treatments (ESTs). The critique refers, for example, to an exclusive focus on symptom improvement while neglecting psychosocial functioning, to limited generalizability of results from research settings to clinical practice, to requiring only two RCTs for demonstrating efficacy or to neglecting design flaws or researcher allegiance (3). For these reasons a new model has been proposed to update the criteria for empirically supported psychotherapy, taking several aspects critically discussed into account (3). This update emphasizes a focus on systematic (quantitative) reviews rather than on individual studies, on the quality of studies, on clinical significance in addition to statistical significance, on long-term outcomes in addition to short-term efficacy, on functional or other health-related outcomes in addition to symptoms, or on generalization to non-research settings (3). In addition, adverse events are taken into account. Categorical diagnoses are de-emphasized, syndromes of psychopathology and diagnostically complex patients are emphasized. Another focus is put on mechanisms of psychopathology and therapeutic change (3). The new model proposes to evaluate candidate treatments along these criteria. It suggests to appraise the available evidence by an expert committee, using the Grading of Recommendations Assessment, Development, and Evaluation (GRADE) system assessing the quality of evidence, the extent to which benefits exceed potential harms and the strength of recommendations that can be made (4–7). In the GRADE system the evidence is judged as “high quality,” “moderate quality” “low quality” or “very low quality” (4–7).

For high quality evidence, the proposed new model for ESTs requires a “wide range” of studies with no major limitations, small heterogeneity and a narrow confidence interval for the summary estimate (3). Here the group proposing the new model for ESTs deviated considerably from the original approach of the GRADE group who considered “one or more well-designed RCTs yielding consistent directly applicable results” as necessary for high quality evidence [(6), p. 178]. Moderate quality evidence is defined in the new model for ESTs by “a few” studies of which some show limitations but no “major flaws” and a wide confidence interval for the summary estimate [(3), p. 13]. Here again, the proposed new model deviates in an important aspect from the original approach of the GRADE group which required “important” limitations for a designation of evidence as being of moderate quality [(6), p. 178]. Compared to the original GRADE approach the deviations introduced by the new model for psychotherapy may result in fewer treatments designated as being of high and moderate quality. Thus, the deviations from the original internationally established GRADE system may have important consequences. They are, however, not well justified.

In a further step, the original GRADE system results in “strong” or “weak” recommendations for a treatment (4–6). In the new model of ESTs a third category is proposed, i.e., a “very strong” recommendation (3), again, however, without a convincing justification. A “very strong” recommendation requires high quality evidence, clinically meaningful effects on symptoms and functioning, short-term and long-term effects ≥ three months, low risk of harms, reasonable costs, and at least one study on effectiveness (see Table 1) (3).

**Table 1:** Checklist for updated criteria of empirically supported treatments (3).

**Mental disorder**			
**treatment**	**yes**	**no**	**?**
Criteria for committee members fulfilled: Expertise, conflicts of interest, diversity etc.			
**1. Systematic review (SR)**			
Are there recent SRs for PDT conducted within the past 2 years or older reviews if results are robust?			
Is there a sufficient (conceptual) homogeneity between treatments?			
Are PICOTS clearly defined in the systematic review?			
Is the quality of the systematic review(s) sufficient?			
Is the quality of studies sufficient?			
Was treatment fidelity demonstrated (manuals, qualified therapists, monitoring, treatment integrity empirically assessed)?			
Is there no serious risk of bias in individual studies?			
Are there clinically meaningful effects in symptom improvement?			
Are there clinically meaningful effects in functioning?			
Is statistical heterogeneity acceptable (low or moderate)?			
Are there long-term effects (≥ 3 months) in addition to short term effects?			
Are there no clinically significant differences in efficacy compared to other active therapies?			
Were syndromes examined, not only categorical diagnosis?			
Was generalizability demonstrated (effectiveness, complex patients, usual therapists/therapy)?			
Is there evidence for ingredients, mechanisms of change?			
Are the data on harms justifying to apply the treatment?			
Is the treatment cost-effective?			
Is there a positive balance of benefits in relation to costs and harms?			
**2. GRADE according to the new model of ESTs** (3)			
**2.1 Quality of evidence**			
High quality evidence: a wide range of studies with no major limitations, small variation between studies and narrow confidence intervals.			
Moderate quality: a few studies of which some have limitations but no major flaws and wide confidence intervals.			
Low quality: studies with major flaws, important variation between studies, and very wide confidence intervals.			
**2.2 Treatment recommendations**			
Very strong: High quality evidence, clinically meaningful effects on symptoms and functioning, short-term and long-term effects ≥three months, low risk of harms, reasonable costs, ≥ 1 study on effectiveness.			
Strong evidence: Moderate to high quality evidence, clinically meaningful effects in symptoms or functioning, positive balance of benefits, costs, harms, no effectiveness study required.			
Weak evidence: Low or very low quality of evidence for meaningful effects in symptoms and/or functioning or only statistical statistically significant effects, unclear whether gains warrant costs and harms.			

PICOTS, Patients, interventions, comparators, outcomes, timeline, setting.

Following the previous criteria of ESTs (1, 2), the empirical status of psychodynamic therapy (PDT) was assessed in several reviews (8–12). The revised set of criteria for ESTs, however, has not yet been applied to research available for PDT. Thus, it is not clear to what extent PDT fulfills the updated criteria for ESTs in specific mental disorders. However, as the Task Force on Promotion and Dissemination of Psychological Procedures put it some years ago it is critical to investigate whether PDT fulfills the updated criteria “if this clinically verified treatment is to survive in today's market” (13). Today, this statement is more true than ever. For this reason, we will carry out a systematic evaluation of PDT in common mental disorders applying the revised criteria of ESTs (3). The criteria are summarized in Table 1.

Wherever possible, we will apply an integrative approach for the various forms of PDT. For many types of PDT it has been shown that the commonalities in theory and techniques outweigh the differences and allow for the development of unified protocols (14–16). Unified psychodynamic protocols focus on shared ingredients or mechanisms, representing a “mechanistical approach” (14–16). A unified transdiagnostic approach corresponds well to the updated model for ESTs that encourages a focus on core dimensions of pathology and treatments which may reduce “the EST movement's reliance on a large number of treatment manuals” and may lead to a much simpler and “more practitioner-friendly system” [(3), p. 10]. Following the updated model for ESTs (3), we will evaluate the empirical status of PDT in common mental disorders along the criteria proposed by the new model (Table 1) (3). In a next step, we will grade the evidence and assess the strength of recommendations following GRADE (3–6).

## Review panel

The authors of the planned review fulfill the criteria proposed for the new model of ESTs (3), that is (a) a broad range of documented expertise, (b) disclosure of actual and potential conflicts of interest, (c) maintaining a climate of openness, (d) using clearly defined procedures and methods as described in the study protocol.

## Methods

### Searches

We will search PubMed and PsycINFO and individual records of the Cochrane Library for systematic reviews, meta-analyses and individual randomized controlled trials (RCTs) on the efficacy of PDT in common mental disorders in adults published between 2012 and August 2022. Search terms will contain review, meta-analy^*^, metaanaly^*^ or random^*^ combined with psychodynamic or dynamic or psychoanalytic therapy. Additionally, a regularly updated comprehensive list containing RCTs of psychodynamic treatments will be consulted (www.researchgate.net/publication/317335876), and hand searches for systematic reviews and individual studies in journal articles and textbooks will be carried out. With regard to the setting in which PDT is applied, both face-to-face and internet PDT will be included as well as individual and group therapy. Furthermore, we will search for systematic reviews and for individual randomized and open studies on ingredients and mechanisms of change of PDT, for effectiveness studies carried out under real-world conditions, for studies of adverse events and for studies on cost-effectiveness of PDT (3). Additional search terms will be *mechanisms of change, curative factors, process-outcome, cost-effectiveness*. At least two reviewers will independently screen the results of the database search for relevant meta-analyses and individual studies. If the title and abstract of an study contain sufficient information to determine that an article does not meet the inclusion criteria specified below, that article will be rejected. In a next step, full texts of all studies possibly relevant for inclusion will be retrieved. Disagreements about the inclusion of a meta-analysis or study will be solved by consensus or consulting a third expert. The search results will be documented in a PRISMA flow chart.

### Type of study to be included

With regard to efficacy, we will follow the proposed revised criteria for empirically supported treatments by focusing on systematic (quantitative) reviews of randomized controlled trials (RCTs) of PDT in common mental disorders in adults (3). As suggested, the focus will be on recent systematic (quantitative) reviews published in the past 2 years (3). Older reviews will be eligible if the results are robust, that is it is unlikely that recent research would affect the empirical status of a treatment (3). Meta-analyses are required to test PDT against a comparator, including waiting list, treatment as usual (TAU), pill or psychological placebo, pharmacotherapy or another form of psychotherapy (3). Results will be evaluated per disorder and comparator. For studies of mechanisms of change, both RCTs and open studies will be included. If no systematic reviews on a specific research question are available (e.g., on cost-effectiveness), individual RCTs and open studies will be included as well.

### Condition or domain being studied

Common mental disorders in adults according to DSM or ICD (excluding organic mental disorders), including depressive disorders, anxiety disorders, trauma- and stressor-related disorders, dissociative disorders, obsessive-compulsive disorders, eating disorders, somatic symptom disorders, attention deficit/hyperactivity disorder, substance related disorders, personality disorders, bipolar disorders, and schizophrenia spectrum disorders are eligible. As mental disorders rarely occur as “pure” disorders, we will evaluate if comorbidity was taken into account (e.g., by sensitivity analyses) and possibly affected outcome.

In addition, complex mental disorders will be included, defined as chronic disorders, highly comorbid mental disorders and disorders associated with personality disorders.

### Participants/population

Adult participants (≥18 years) meeting diagnostic criteria for any of the mental disorders listed above.

### Intervention(s), exposure(s)

The review will focus on psychodynamic therapy (PDT). PDT includes a family of psychotherapeutic approaches which focus on the therapeutic relationship, expression of emotion, exploration of defensive (avoidance) patterns, identification of recurring themes, discussion of past experiences, as well as on interpersonal issues (17). Psychodynamic treatments, especially short-term variants, include the development of a therapy focus addressing a patient's presumed unconscious conflicts, internalized object relations or structural impairments (18). PDT operates on an interpretive-supportive continuum (17). The use of more interpretive or supportive interventions depends on the patient's needs (17, 19, 20). While interpretive interventions enhance the patient's insight about repetitive conflicts sustaining his or her problems, supportive interventions aim at strengthening abilities (“ego-functions”) that are not accessible to the patient due to acute stress or insufficient development (18). For most common mental disorders treatment manuals are available (10).

### Comparator(s)/control

Meta-analyses on efficacy are required to test PDT against any of the following comparators, i.e., waiting list, treatment as usual (TAU), pill or psychological placebo, pharmacotherapy or another form of psychotherapy (3). For each disorder results will be evaluated per control condition, that is for comparisons with unspecific controls (TAU, placebo, waiting list) and for comparisons with active treatments (psychotherapy or pharmacotherapy).

### Context

Outpatient or inpatient treatment.

### Outcome(s)

#### Primary outcome(s)

As critical (primary) outcome (21) we will use effect sizes in disorder-specific target symptoms post-therapy assessed by validated scales, that is, for example, depressive symptoms in depressive disorders or anxiety symptoms in anxiety disorders. In addition to statistical significance, clinical significance of effect sizes will be assessed. If presented by the authors of the included meta-analyses, data of high-quality studies and data corrected for small sample size/publication bias or outliers will be preferably included and interpreted to present the best evidence available.

#### Secondary outcomes

Improvements in functioning, quality of life or other health related outcomes, long-term effects, rates of response and remission as assessed in the included meta-analyses, adverse outcomes, effectiveness under real-world conditions, cost-effectiveness, and data on ingredients and mechanisms of change of PDT will be included when available (3).

If the evidence differs between primary (critical) outcomes and other outcomes such as side effects or costs, GRADE regards efficacy outcomes as the most important on most occasions and suggests that guideline panels can base their rating of the quality of evidence exclusively on data on efficacy [(6), p. 180]. If side effects are not severe, we will follow this suggestion.

### Data extraction (selection and coding)

For retrieving details of included meta-analyses and individual studies, a data extraction form will be used. At least two authors will independently extract the results (type of disorder, number of included RCTs, number of participants, risk of bias, adverse events, effect sizes, 95% confidence intervals, and heterogeneity).

### Assessing the quality of included meta-analyses

The quality of the included meta-analyses will be assessed independently by two raters using a checklist developed by Aromataris et al. (22), using the first nine items which refer to quality.

#### Quality of primary studies

Ratings of study quality and/or of risk of bias reported by the included meta-analyses will be evaluated. If data on risk of bias are not reported by the authors of the meta-analyses or individual studies, we will carry out ratings of risk of bias for the included studies. For this purpose we will use the four criteria proposed by Cuijpers et al. (23), that is adequate random sequence generation, allocation concealment, blinding of assessors and/or use of self-report measures only and use of intent-to treat-analysis to take incompleteness of data into account. Ratings will be made independently by two raters, discrepancies will be solved by consensus or by inclusion of a third rater. In addition, treatment fidelity will be evaluated including the use of treatment manuals, experienced and trained therapists, monitoring of therapy and empirical assessment of treatment integrity (3). With regard to the quality of studies on mechanisms of change we will follow the suggestions by Crits-Christoph and Connolly Gibbons (24), e.g., applying multilevel modeling of therapist vs. patient contributions, approaches to understand potential causality, and testing of specificity of effects.

### Strategy for data synthesis

Along the updated criteria of ESTs (3), PDT will be evaluated for each of the included common mental disorders regarding several parameters (Table 1), including effect sizes in target symptoms and functioning, clinical significance of effect sizes, study quality, risk of bias, results in follow-up studies, adverse events, cost-effectiveness, generalizability of results (results under real-world conditions), evidence for ingredients and mechanisms of change. Quality of evidence will be rated according to GRADE (6). In a next step the strength of treatment recommendations will be assessed (3, 7).

### Analysis of subgroups or subsets

If appropriate, results of subgroups or subsets will be reported, e.g., for treatment duration or dose, minority groups or high quality studies.

## Data availability statement

The original contributions presented in the study are included in the article/supplementary material, further inquiries can be directed to the corresponding author.

## Author contributions

All authors listed have made a substantial, direct, and intellectual contribution to the work and approved it for publication.

## Conflict of interest

The authors declare that the research was conducted in the absence of any commercial or financial relationships that could be construed as a potential conflict of interest.

## Publisher's note

All claims expressed in this article are solely those of the authors and do not necessarily represent those of their affiliated organizations, or those of the publisher, the editors and the reviewers. Any product that may be evaluated in this article, or claim that may be made by its manufacturer, is not guaranteed or endorsed by the publisher.
